# Meibum Lipid Saturation Related to Dry Eye, Age, and Sex Using Nuclear Magnetic Resonance Spectroscopy

**DOI:** 10.1167/iovs.67.3.60

**Published:** 2026-03-30

**Authors:** Djihane Machane, Muhammed Shah Shaji, Douglas Borchman, Vance Jaeger

**Affiliations:** 1 Department of Ophthalmology and Visual Sciences, University of Louisville, Louisville, Kentucky, United States; 2 Department of Chemical Engineering, University of Louisville, Louisville, Kentucky, United States

**Keywords:** age, dry eye, lipid, meibum, meibomian gland dysfunction, nuclear magnetic resonance, saturation

## Abstract

**Purpose:**

The degree of unsaturation in hydrocarbon chains (HCunsat) is likely to play an important role in determining the stability, order, and fluidity of lipids in the tear film lipid layer. The HCunsat of meibum lipids was investigated in relationship with age, sex, dry eye, Sjögren's syndrome, Parkinson's disease, and treatment for allogeneic hematopoietic stem cell transplantation.

**Methods:**

Meibum was expressed and HCunsat was measured using ^1^H-NMR resonances at 5.33 and 2.00 ppm. HCunsat levels were normalized to the level of wax esters, cholesteryl esters, and hydrocarbons.

**Results:**

HCunsat levels, normalized to lipid esters, were found to be 20% to 40% lower in individuals aged >50 years compared with those <50 years (*P* < 0.05) and showed a linear age-dependent decrease with age (*P* < 0.001). No substantial changes were observed with sex and race, except for a 24% reduction in a small Hispanic group (*P* = 0.0078) compared with Caucasians. A cohort with dry eye had a ∼13% lower level of HCunsat, whereas cohorts susceptible to dry eye such as those with Sjögren's syndrome, Parkinson's, and graft-versus-host disease had a ∼35% lower HCunsat levels compared with a cohort without dry eye (*P* < 0.05). Experimental deviations were minimal (<4%),

**Conclusions:**

Meibum HCunsat decreases with age and is lower with dry eye, Sjögren's syndrome, and Parkinson's disease cohorts compared with a cohort without dry eye. This decrease in HCunsat is expected to contribute to lipid aggregation, decreased lipid spreading, and meibomian gland outflow. ¹H-NMR was effective for quantifying major meibum lipids.

The tear film is a thin, 2.0- to 5.5-µm layer^1^^–^^5^ on the ocular surface. A 40- to 74-nm layer of lipid^6^^–^^8^ called the tear film lipid layer (TFLL) sits atop the tear aqueous layer. It helps tears to spread evenly and keeps the tear film stable by decreasing surface tension.^9^^–^^11^ About 80% of the TFLL comes from meibum, which is produced by the meibomian glands in the eyelids.^12^ The other 20% is likely to come from sebaceous glands, which contribute to TFLL phospholipids and squalene.^13^ Meibum is composed of >90% nonpolar lipids primarily wax esters (WE) and cholesterol esters (CE). More than 236 lipid species have been identified in tears.^12^

Dry eye disease (DED) is a common eye condition where the tear film becomes unstable, leading to discomfort and vision problems.^14^^–^^17^ Meibomian gland dysfunction (MGD) is a key factor related to DED.^18^^,^^19^ TFLL compositional changes contribute to DED.^20^

The degree of unsaturation (more double bonds) in the hydrocarbon chain (HCunsat) is the primary factor influencing the phase transition temperature and order (fluidity) of lipids.^21^^,^^50^ A higher level of HCunsat is linked to increased hydrocarbon chain disorder, whereas a lower degree results in more ordered, rigid hydrocarbon chain structures.^20^^–^^23^ Maintenance of the meibum lipid structure is crucial for tear film stability, because more ordered lipids, which are stiffer and more saturated, can cluster together, blocking the meibomian glands and preventing the proper spreading of meibum across the ocular surface.^20^ Decreasing the HCunsat in human meibum and tear lipids resulted in thicker, less elastic lipid films, with effects proportional to the level of HCunsat.^21^^–^^23^ An infrared spectroscopy study of human meibum suggests that individuals with MGD have a higher level of HCunsat,^24^ leading to increased lipid order^25^ and decreased flow. However, another study using Raman spectroscopy did not support this observation, perhaps owing to the small number of samples (eight without and five with DED), the large scatter in the data, and the resolution of the CH_2_ twisting and =C–H in-plane deformation bands.^26^ Meibum samples from donors without DED and with mild-to-moderate or severe MGD, were recently analyzed using ^1^H-NMR.^27^ HCunsat was reduced with increasing severity of MGD suggesting that “HCunsat contributes to the pathophysiology of MGD.”^27^

Age-related changes in human meibum may affect the stability of the tear film and its lipid composition. With aging, the HCunsat of human meibum lipid increased, leading to a decrease in hydrocarbon chain order, cooperativity, and phase transition temperature.^22^ The most substantial changes in these parameters occurred between the ages of 1 and 20 years and, perhaps influenced by hormonal factors during adolescence, and are likely to contribute to the development of DED.^22^

Sjögren's syndrome (SS) is a chronic autoimmune condition primarily affecting women, causing lymphocytic infiltration of exocrine glands that significantly reduces glandular secretions.^28^^,^^29^ This condition typically presents as DED and dry mouth, along with fatigue and joint pain, which can significantly impact a patients' quality of life. MGD associated with SS^30^^–^^32^ is likely to alter the TFLL, which plays a critical role in tear stability.^20^^,^^33^^–^^35^ Spectroscopic analysis of meibum lipid composition in SS provided lipid-related mechanisms that contribute to tear film instability and DED.^36^

Changes in the TFLL were observed with MGD associated with Parkinson's disease (PD) that may lead to DED.^37^ Meibum is more ordered (rigid) with PD compared with meibum from age-matched donors without DED. It has been suggested that structural rigidity can block glands and inhibit the spreading of the TFLL after a blink.^20^^,^^33^^–^^35^

In graft-versus-host disease, patients undergoing allogeneic hematopoietic stem cell transplantation (AHSCT) frequently suffer from severe DED accompanied by more ordered and rigid meibum lipids compared with controls without DED,^38^^–^^40^ which could contribute to tear instability. As with PD and SS, a decrease in fluidity may cause a blockage in the meibomian glands and instability in the tear film. Additionally, lower cooperativity in lipid phase transitions suggests a more heterogeneous lipid environment in these patients.

Because meibum lipid stiffness may contribute to tear film instability, and HCunsat relates directly to lipid stiffness,^20^ in the current study, the relationship between meibum HCunsat and age, sex, SS, PD, DED, and AHSCT treatment, was measured.

Because an understanding of how different demographics and medical conditions influence meibum HCunsat may provide insights into the role of HCunsat and tear film instability, the current study embarked on a retrospective analysis of published ^1^H-NMR spectra of human meibum lipid using novel equations and resonances to measure lipid HCunsat.^27^

## Methods

Deuterated chloroform and glass NMR tubes were purchased from Sigma-Aldrich (St. Louis MO, USA). We purchased 9-mm microvials with Teflon caps from Microliter Analytical Supplies Ind. (Suwanee, GA, USA).

The current study is a retrospective analysis of published ^1^H-NMR spectra of human meibum lipids^13^^,^^20^^–^^23^^,^^38^^–^^50^ using novel equations and resonances^27^^,^^42^ to measure HCunsat. All study procedures were approved by the University of Louisville Institutional Review Board (#11.0319, August 2016). The research followed the ethical guidelines outlined in the Declaration of Helsinki. Participants were recruited through the Kentucky Lions Eye Center, the Robley Rex VA Medical Center, and the James Graham Brown Cancer Center, all located in Louisville, Kentucky. Informed consent to participate in the study was obtained from all participants. For the cohort without DED (Cn), 50 samples from a previous study^22^ were analyzed alongside 89 additional samples included, collected, and measured in the current study. For the DED cohort (C_DED_), 41 samples from a previous study^22^ were analyzed alongside 27 additional samples included, collected, and measured in the current study.

### Clinical Analysis

Both eyes were analyzed from most donors. Participants were assigned to the Cn when the patient's had no history of bone marrow transplantation and their meibomian gland orifices showed no evidence of keratinization or plugging with turbid or thickened secretions and no dilated blood vessels were observed on the eyelid margin. The participants did not recall having dry eye symptoms. Participants were assigned to the cohort C_AHSCT_ if they had undergone AHSCT. Patients in the cohort with ACHSCT (C_ACHSCT_) underwent a full ophthalmic eye examination using a slit lamp. Participants with PD were assigned to the cohort C_PD_ and those with SS were assigned to the cohort C_SS_. Participants with DED were clinically diagnosed as previously described^48^ and were assigned to the cohort C_DED_.

### Collection of Meibum and NMR Analysis

Meibomian glands were expressed as described for the cohorts: Cn, C_DED_,^37^ C_AHSCT_,^38^^–^^41^ C_PD_,^37^ and C_ss_.^36^ All four eyelids were expressed, and approximately 0.5 mg of meibum lipid was collected per individual for direct spectroscopic study. The expressate was collected with a platinum spatula under a slit lamp and the meibum was immediately dissolved into 0.5 mL of deuterated chloroform (Sigma-Aldrich) in a 9-mm microvial with a Teflon cap (Microliter Analytical Supplies Ind.). These samples were immediately stored on dry ice under argon gas and transferred to a facility where they were kept at −20°C under argon gas for subsequent ^1^H-NMR analysis. Analyses were performed within 3 weeks of collection of the sample.

### ^1^H-NMR Analysis

Vials containing samples were sonicated in an ultrasonic bath (Branson Ultrasonics, Sterling Heights, MI, USA) for 10 minutes. The resulting solution was transferred into a glass NMR tube (Sigma-Aldrich) for immediate analysis. Most of the meibum from the Cn were analyzed using a700-MHz spectrometer (Varian, Lexington, MA, USA) at 25°C. The spectrometer that was equipped with a 5-mm ^1^H[^13^C/^15^N] ^13^C-enhanced pulsed field gradient cold probe (Palo Alto, CA, USA).

The resonance of CDCl_3_ at 7.24 ppm was used to verify the chemical shift values of the samples and standard. ^1^H-NMR spectra were measured with a minimum of 1250 scans with a pulse width of 45° and a relaxation delay of 1.000 seconds. This process requires approximately 1.5 hours per sample to ensure a high signal-to-noise ratio.

Samples measured using the 500 MHz instrument (Inova-500 spectrometer; Varian, Lexington, MA, USA) used the following parameters: 800 scans were acquired with a spectral width of 15 parts per million (ppm), 60° pulse, 4-K data points, a 1.0-second delay time, and a 2.049-second acquisition time at 25°C.

Phasing, curve fitting, and integration of the spectral data were performed using GRAMS 386 software (Galactic Industries Corp., Salem, NH, USA). After analysis, the samples were returned to their original 9-mm microvials for storage.

### Measurement of Hydrocarbon Chain Unsaturation

**Figure 1.**
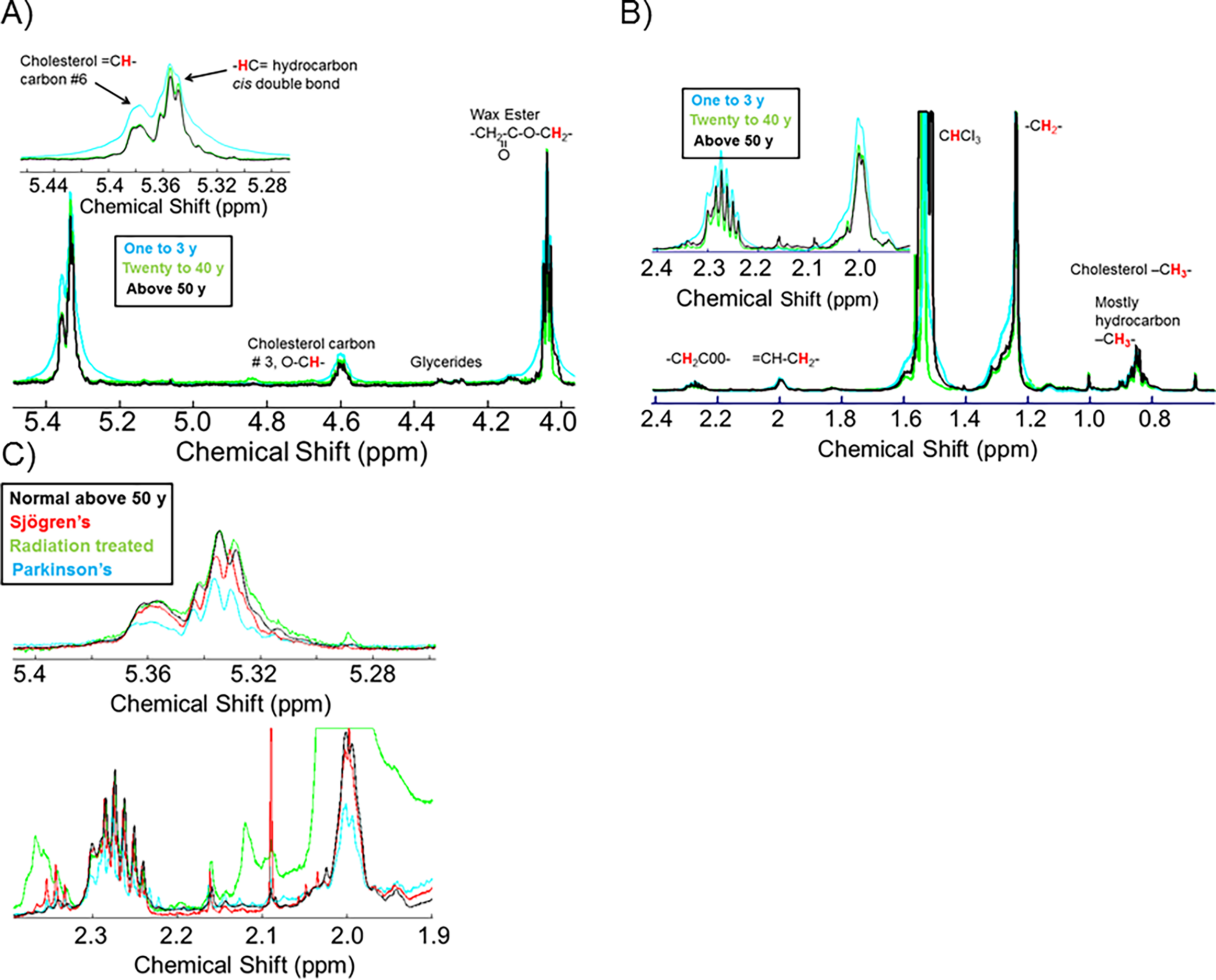
Average ^1^H-NMR spectra of regions used to measure lipid unsaturation. Spectra were normalized to have the same WE intensity at 4 ppm. (**A**) Ester and double bond regions. (**B**) double bond and methylene regions. (**A** and **B**) (*blue*) cohort of participants ranging in age from 1 to 3 years old; (*green*) cohort of participants ranging in age from 20 to 40 years old; (*black*) cohort of participants older than 50 years. Note in the inset spectra in A and B how the –HC= intensity at 5.33 ppm and the CH-C**H**_2_- intensity at 2 ppm was lower with age. (**C**) Note how the intensities of the –HC= intensity at 5.33 ppm are lower for the Sjögren's (*red*), PD (*blue*), and radiation treated (*green*) cohorts are lower compared with the normal cohort (*black*). The large resonance at 2 ppm for the radiation treated (*green*) cohort is unlikely due to unsaturation but rather a moiety resulting from the treatment.

To evaluate the HCunsat, the resonance observed at 5.33 ppm, associated with cis –CH= double bonds, was analyzed (Fig. 1A). The resonance intensity of the =CH at 5.36 ppm from the proton on carbon #6 of cholesterol was subtracted from the combined intensity of the signals at 5.36 and 5.33 ppm. For CE analysis, the resonance intensity at 5.33 ppm was normalized to the sum of the WE and CE resonance intensities and the hydrocarbon resonances (Fig. 1A).

To measure HCunsat, the resonance at 2.0 ppm, assigned to the =CH–CH_2_ moiety were used (Fig. 1C). For the normalization of HCunsat to the resonances at 4.0 ppm for WE (Fig. 1A) and at 1.0 and 0.63 ppm for the CH_3_ moieties of CE and the hydrocarbon chain CH_2_ resonances at 2.29, 2.0, and 1.25 ppm were used (Fig. 1B). The later resonances correspond with protons associated with fatty acids (–CH_2_–COO–), =CH–CH_2_, and –CH_2_– moieties, respectively.

The following equations were used:

(1) $$\begin{array}{ccc} & & \;\mathrm{Runsat}/\left(\mathrm{WE}\;+\;\mathrm{CE}\right)\;\\ & & \;=\left(I_{5.33}/2\right)\;/\;\left[\left(I_{1.0}+I_{0.63}\right)\;/\;6\;+\;\left(I_{4.0}\right)\;/\;2\right] \end{array}$$

(2) $$\begin{array}{c} \mathrm{Runsat}/\mathrm{hydrocarbons}=\left(I_{5.33}/2\right)/\left[\left(I_{2.29}+I_{1.25}+I_{2.0}\right)/2\right] \end{array}$$

(3) $$\begin{array}{c} \mathrm{Runsat}/\left(\mathrm{WE}+\mathrm{CE}\right)=\left(I_{2.0}/4\right)/\left[\left(I_{1.0}+I_{0.63}\right)/6+\left(I_{4.0}\right)/2\right] \end{array}$$

(4) $$\begin{array}{c} \mathrm{Runsat}/\mathrm{hydrocarbons}=\left(I_{2.0}/4\right)/\left[\left(I_{2.29}+I_{1.25}+I_{2.0}\right)/2\right] \end{array}$$

The variability in HCunsat from a single Caucasian 53-year-old participant without dry eye was determined at the start of the collection by collecting meibum and measuring HCunsat once, then, after 15 years, five times 1 week apart.

### Statistical Analyses

The Student's *t* test was used to measure *P* values for averages. Critical values of the Pearson product-moment correlation coefficient were used to measure *P* values to determine linearity. Data are presented as averages with the standard error of the mean.

## Results

### Donor Demographics

Exactly 239 meibum samples were collected from a diverse group of donors, categorized by race, sex, and age. Donor demographics are provided in Table 1.

**Table 1.** Participant Demographics

Cohort	No.	Male (%)	Caucasian (%)	Asian (%)	Hispanic (%)	Black (%)	Unknown (%)	Average Age^*^ (Years)
No dry eye	99	64	78	6	4	9	3	27 ± 2
Dry eye	30	48	86	0	4	10	0	30 ± 2
No dry eye, 500 MHz	40	82	75	17.5	2.5	2.5	2.5	30 ± 4
Dry eye, 500 MHz	38	77	60	0	23	11	26	66 ± 3
SS	7	0	57.1	0	14.3	28.6	0	51 ± 4
PD	10	100	100	0	0	0	0	62 ± 16
PD with dry eye	3	33	100	0	0	0	0	66 ± 5
Graft-versus-host disease	12	58.3	91.7	0	0	8.3	0	52 ± 11

* Averaged and standard error of the mean. We used 700-MHz instruments unless indicated.

### ^1^H-NMR Spectra

The average ^1^H-NMR spectra differed among the cohorts in the regions related to HCunsat (Fig. 1). Note that the –HC= intensity at 5.33 ppm and the CH–CH_2_– intensity at 2 ppm was lower with age (Fig. 1A, 1B) and PD and SS (Fig. 1C). Spectra were normalized to have the same WE intensity at 4 ppm before averaging.

### Unsaturation Levels With Age

**Figure 2.**
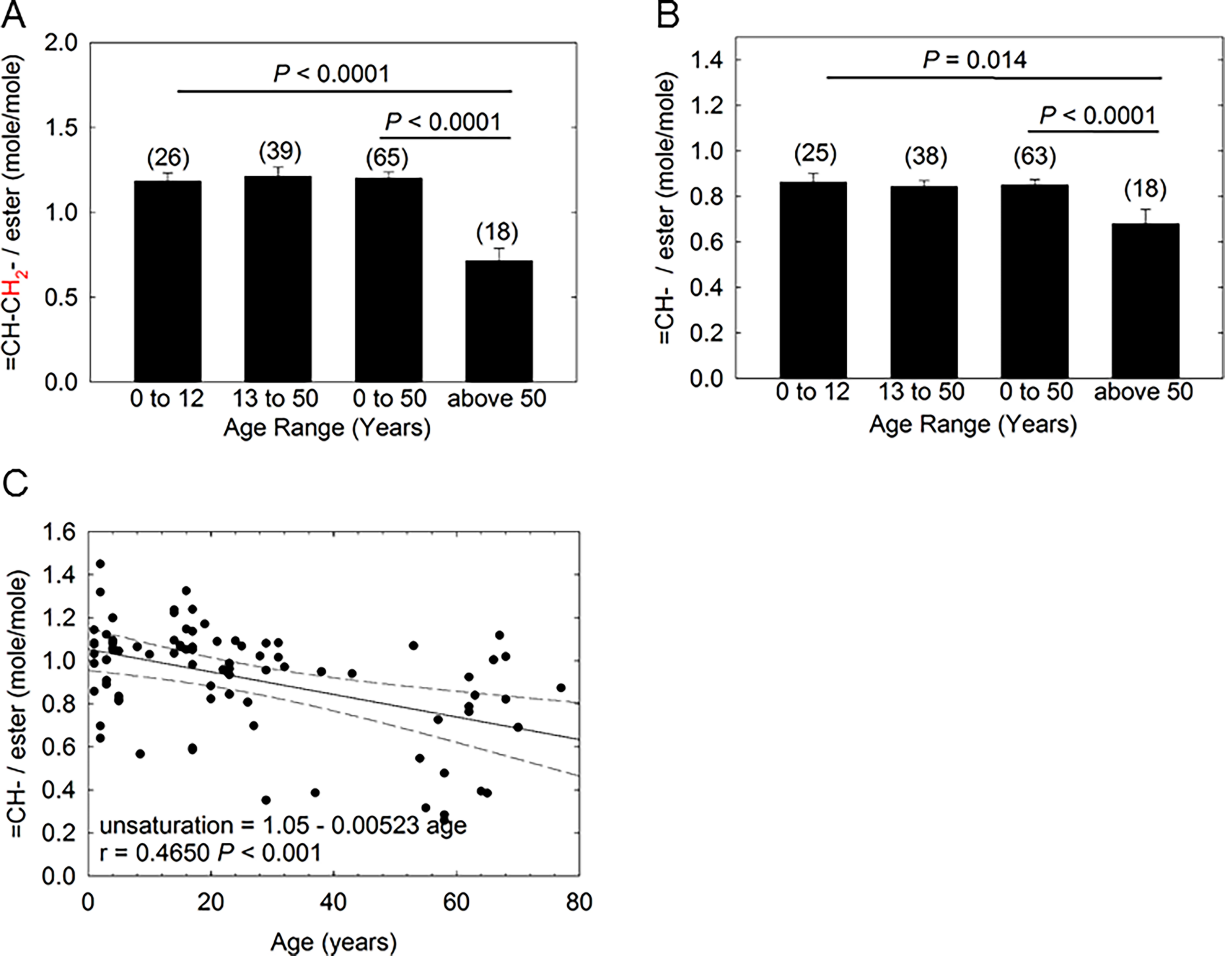
Lipid saturation measured from ^1^H-NMR spectra of human meibum from participants without dry eye. (**A**) Saturation was measured relative to the amount of CE and WE using the Equation 3. (**B**) Saturation was measured relative to the amount of CE and WE using the Equation 1. (**C**) The *y* axis is the average of A and B. Numbers in parenthesis are the number of samples. The Student's *t* test was used to measure *P* values in A and B. Critical values of the Pearson product-moment correlation coefficient were used to measure *P* values in C. Error bars are the standard error of the mean. (—-) 95% confidence limits.

The intensity of the resonances for HCunsat (2.0 ppm or 5.33 ppm) relative to the sum of the intensities of the WE and CE resonances or to the hydrocarbon resonances were measured using Equations 1 and 3 and Equations 2 and 4, respectively. HCunsat normalized to the level of esters were 20% to 40% lower (*P* < 0.05) when comparing the age group aged <50 years and >50 years (Fig. 2A, 2C). The average Values of HCunsat using Equations 1 and 3 decreased with age linearly, *P* < 0.001 (Fig. 2C). The linear decrease in HCunsat with age was also evident when HCunsat was measured using Equation 1 alone, (=CH-CH_2_/ester = 1.26 – 0.00735 age, r = 0.5428, *P* < 0.001), or when HCunsat was measured using Equation 3 alone, (=CH/ester = 0.90 – 1.97 age, r = 0.2653, *P* = 0.26) where r is the correlation coefficient.

HCunsat relative to the amount of CH_2_ moieties did not change with age (*P* > 0.05). The =C–/CH_2_ measured on all of the age groups using Equations 2 and 4 were in excellent agreement, 0.036 ± 0.002 (*n* = 66) and 0.038 ± 0.002 (*n* = 70), respectively.

The average Runsat/(WE + CE) for Cn was similar (*P* = 0.006), 0.97 ± 0.12 (*n* = 46) using the 500-MHz spectrometer compared with using the 700-MHz instrument. However, Runsat/(WE + CE) using the 500-MHz spectrometer was three times more variable compared with the Runsat/(WE + CE) using the 700-MHz and only the 500-MHz resonances using Equation 1 were reliable.

### Runsat/(WE + CE) Levels With Sex and Race

No significant differences (*P* > 0.05) in Runsat/(WE + CE) were observed with sex and, race except for a small group of Hispanics, which were 24% lower (*P* = 0.0078) compared with Caucasians (Table 2). Regarding race, caution should be observed owing to the small sample size.

**Table 2.** Lipid Levels With Sex and Race

Parameter	Unsaturation Per Ester (Mole:Mole From Equations 1 and 3	*P* Value
Male	0.84 ± 0.03 (54)	vs Female
Female	0.77 ± 0.04 (28)	0.14
Caucasian	0.84 ± 0.02 (68)	vs. Caucasian
Hispanic	0.64 ± 0.16 (4)	^*^0.0078
Asian	1.00 ± 0.03 (5)	0.61
Black	0.83 ± 0.15 (4)	0.10

Values are average ± standard error of the mean.

* Significant difference.

### Unsaturation Levels With SS, PD, and AHSCT

**Figure 3.**
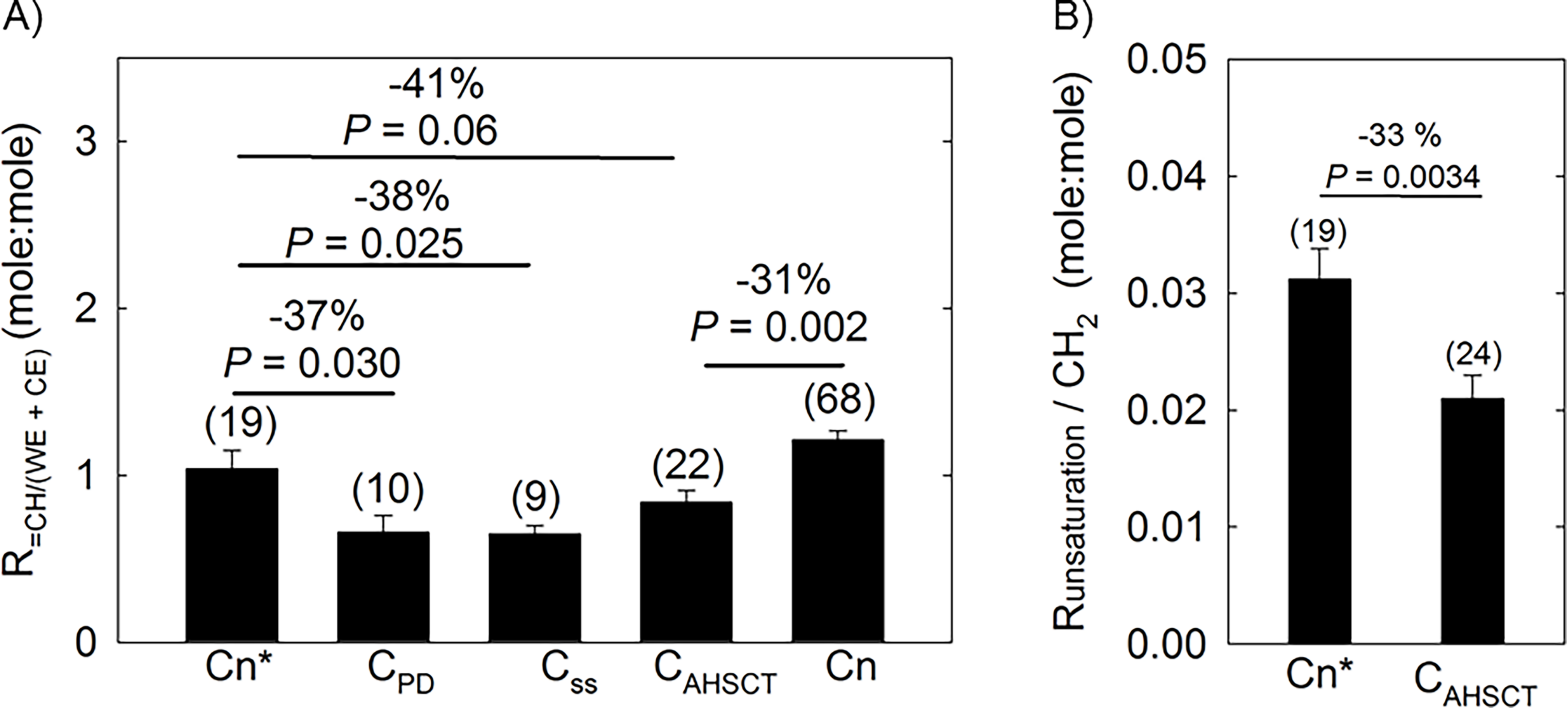
Unsaturation levels from ^1^H-NMR spectra of human meibum from cohorts susceptible to dry eye. (**A**) R_unsaturation/(WE + CE)_ was calculated by averaging the values for each sample using Equations 1 and 3 (Methods). (**B**) R_unsaturation/CH2_ was calculated by averaging the values for each sample using Equations 2 and 4 (Methods). Cn is the cohort without dry eye including all ages. *Cn is the cohort without dry eye age-matched to other cohorts. C_PD_ is the cohort with PD, C_AHSCT_ is a cohort that has undergone allogenic hematopoietic stem cell transplantation, and C_SS_ is a cohort with SS. The Student's *t* test was used to measure *P* values relative to Cn. Error bars are the standard error of the mean.

The average ^1^H-NMR spectra in regions related to HCunsat differed for small groups susceptible to DED (Fig. 1C). Spectra were normalized to have the same WE intensity at 4 ppm before averaging. The large resonance at 2 ppm for the radiation-treated (green) cohort (Fig. 1C) is unlikely owing to HCunsat but rather a moiety resulting from the AHST treatment. Note in (Fig. 1C) how the intensities of the –HC= intensity at 5.33 ppm are lower for the SS (red) and PD (blue) cohorts compared with the normal cohort (black). Two equations were used to measure the intensity of the resonances for HCunsat (2.0 or 5.33 ppm) relative to the sum of the intensities of the WE and CE resonances (Equations 1, 3) or to hydrocarbon resonances (Equations 2, 4). Runsat/_(WE + CE__)_ was significantly lower (31% to 41%; *P* < 0.05) for C_SS_, C_AHSCT_, and C_PD_ compared with the Cn measured by averaging Equations 1 and 3 for each sample (Fig. 3A). Runsat/CH_2_ was significantly lower (33%; *P* < 0.05) for C_AHSCT_ compared with the Cn* measured by averaging Equations 2 and 4 for each sample (Fig. 3B).

The decreases were similar for each equation compared with the average of the two equations (Tables 3, 4). The major conclusion from the above data is that the levels of unsaturation were lower for C_DED_, C_PD_, C_AHSCT_, and C_SS_ compared with Cn (Fig. 3).

**Table 3.** Parameters and Values for R_unsaturation/(WE + CE)_

Cohort, All Using a 700-MHz Instrument	Equation 1	Equation 3	Equations 1 and 3 Averaged
Cn,	0.82 ± 0.02, 85	1.05 ± 0.03, 83	0.93 ± 0.03, 85
C_DED_	0.82 ± 0.07, 30	0.89 ± 0.07, 30	0.84 ± 0.06, 30
Cn*	0.78 ± 0.07, 22	0.89 ± 0.09, 15	0.77 ± 0.05, 26
Css	0.56 ± 0.09, 9	0.74 ± 0.15, 9	0.65 ± 0.05, 9
P_PD_	0.545 ± 0.09, 10	0.77 ± 0.13, 10	0.66 ± 0.10, 10
P_PD_ with dry eye	0.37 ± 0.11, 3	0.46 ± 0.16, 3	0.42 ± 0.13, 3
C_AHSCT_	0.78 ± 0.07, 22	0.89 ± 0.09, 15	0.84 ± 0.07, 22

Values are (mole:mole) ± standard error of the mean, number of samples.

Cn* aged matched for all but C_DED_.

**Table 4.** Parameters and Values for R_unsaturation/CH2_

Cohort, All Using 700 a MHz Instrument	Equation 2	Equation 4	Equations 2 and 4 Averaged
Cn	0.034 ± 0.002, 66	0.038 ± 0.002, 70	0.036 ± 0.002, 66
C_DED_	0.032 ± 0.003, 30	0.033 ± 0.002, 30	0.031 ± 0.002, 30
Cn*	0.024 ± 0.004, 19	0.035 ± 0.003, 19	0.0312 ± 0.0026, 19
Css	0.023 ± 0.005, 9	0.027 ± 0.009, 8	0.030 ± 0.013, 9
P_PD_	0.024 ± 0.004, 10	0.034 ± 0.005, 10	0.029 ± 0.005, 10
P_PD_ with dry eye	0.34 ± 0.01, 3	0.021 ± 0.007, 3	0.027 ± 0.009, 3
C_AHSCT_	0.017 ± 0.008, 13	0.025 ± 0.002, 15	0.021 ± 0.002, 24

Values are (mole:mole) ± standard error of the mean, number of samples.

Cn* aged matched for all but C_DED_.

### Unsaturation Levels With Dry Eye

**Figure 4.**
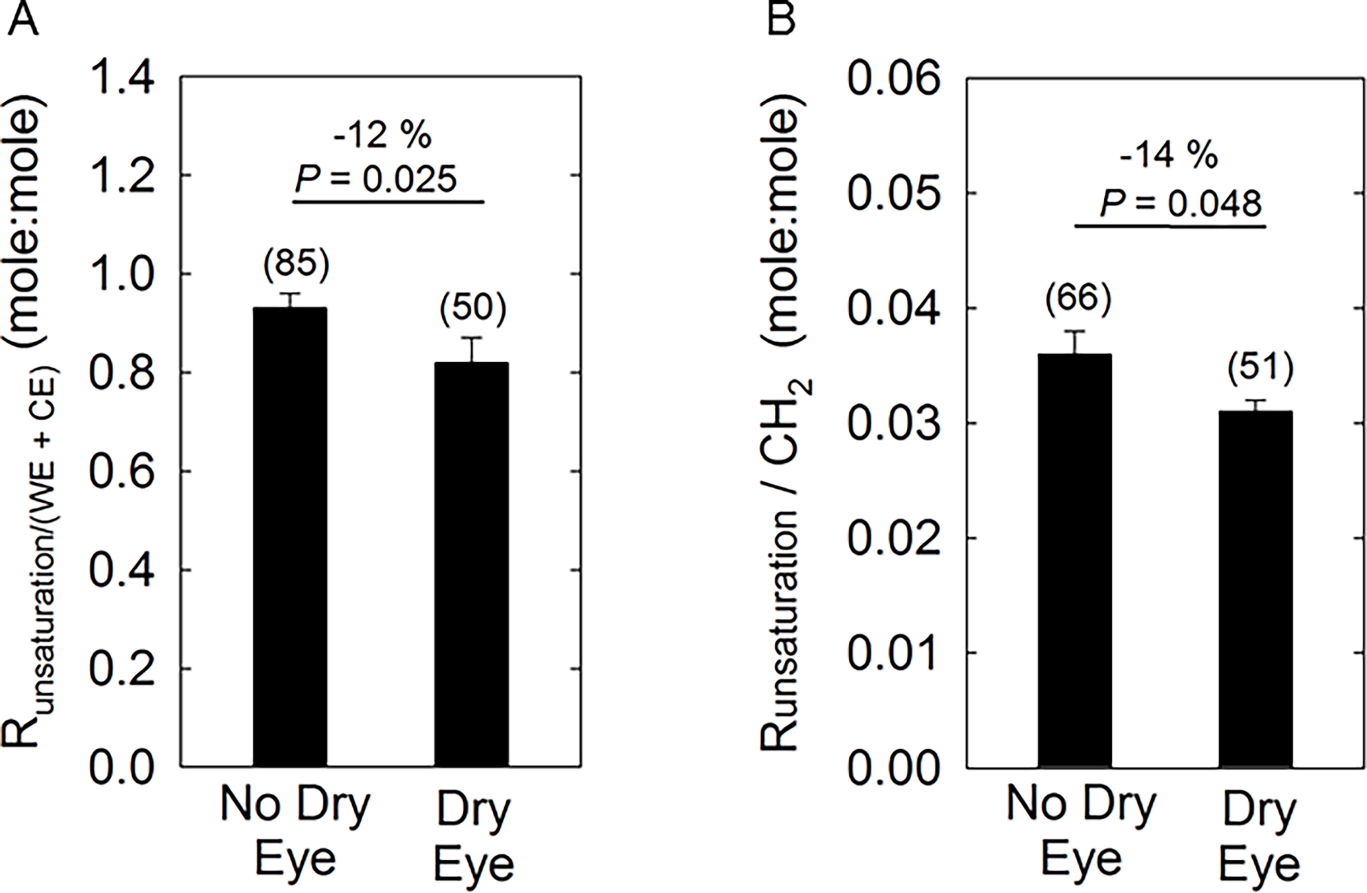
Lipid saturation measured from ^1^H-NMR spectra of human meibum from participants without dry eye. (**A**) Saturation was measured relative to the amount of CE and WE using the equation. (**B**) Saturation was measured relative to the amount of CE and WE using the equation. (**C**) The *y* axis is the average of A and B. Numbers in parenthesis are the number of samples. The Student's *t* test was used to measure *P* values. Error bars are the standard error of the mean.

Runsat/(WE + CE) and Runsat/CH_2_ were calculated for CDED using five different resonances, and the 700-MHz spectrometer. Runsat/(WE + CE) was calculated by averaging the values for each sample using Equations 1 and 3. Runsat/CH_2_ was calculated by averaging the values for each sample using Equations 2 and 4. Equations 1 and 2 used the =CH resonance at 5.33 ppm, which is not completely resolved from the =CH resonance at 5.36 ppm from the proton on carbon #6 of cholesterol. The resonance =CH at 2 ppm is well-resolved and therefore provides a more accurate measurement of unsaturation compared with the 5.36 ppm resonance. However, both the 5.33 and 2.0 ppm resonance data are presented; to avoid bias, Equations 1 and 3 and Equations 2 and 4 were averaged. Runsat/(WE + CE) was 12% to 14% lower for C_DED_ (*P* < 0.05) compared with Cn (Fig. 4). Differences in the values for Equations 1 and 3 or 2 and 4 were <4%.

### Variability From One Individual Over 15 Years

The variability in HCunsat from a male Caucasian 53-year-old participant without DED at the start of the collection was estimated by collecting meibum and measuring HCunsat once, then five times 1 week apart after 15 years. For unsaturation using the 2 ppm/ester and equation 1, the HCunsat levels were 1.25, 1.18, 1.14, 1.18, and 1.25, for samples collected once, then five times, 1 week apart, after 15 years, respectively. The relative deviation using the 5.33 ppm/ester equation 3 was 4%. For unsaturation using the 5.35 ppm/ester equation, the HCunsat, levels were 0.88, 0.84, 0.86, 0.82 0.86, and 0.87 for samples collected once, then five times, 1 week apart, after 15 years, respectively. The relative deviation using the 5.35 ppm/ester equation was 3%. Because the relative deviations in HCunsat for a given age range were much greater than 3% to 4%, they are not likely due to experimental error.

## Discussion

^1^H-NMR can provide a wealth of information regarding meibum lipid composition, including levels of WE, CE, saturation, (O-acyl)-ω-hydroxy fatty acid,^42^ and hydrocarbon chain branching.^43^^,^^45^ Previous ^1^H-NMR studies focused on WE, CE, and hydrocarbon chain branching levels.^36^^–^^50^ The current study involved the analysis of 239 meibum lipid ^1^H-NMR spectra using novel equations and resonances to measure lipid saturation. The major finding of the current study is that HCunsat was lower with age, and for the C_DED_, C_SS_, and C_PD_ C_AHSCT_ compared with Cn. The decrease in HCunsat with age was linear concomitant with tear breakup time from other studies^20^^,^^51^^,^^52^ and a steady increase in blink rate with age.^20^^,^^53^^,^^54^ The lower level of HCunsat for C_DED_ compared with Cn in the current study is in agreement with our recent study^27^ that showed a progressive decrease in meibum HCunsat associated with increasing disease severity, suggesting the change may contribute to the DED. Coupled with our current age-related results and the finding that C_SS_ and C_PD_ C_AHSCT_, who are susceptible to DED and have a low level of HCunsat, suggests that HCunsat may contribute to lower tear breakup times and higher blink rates.

A rationale for the correlations between tear film stability and HCunsat levels with age, SS, and PD disease, and the severity of DED can be made. HCunsat is a key factor affecting the phase transition temperature and fluidity of lipids.^20^^,^^21^^,^^50^ Based on the results of our catalytic saturation study,^22^ a 50% decrease in HCunsat with age (from 0 to 80 years) or with severe DED would cause human meibum to go from 68% disordered to 20% disordered and would increase the phase transition temperature from 30°C to 40°C. Thus, HCunsat accounts for the difference in the lipid order (stiffness) of meibum between individuals with and without MGD.^25^ Indeed, the linear age-related decrease in HCunsat observed in the current study correlates with a linear decrease in the stiffness and increase in elasticity of the TFLL estimated from the inverse of the in-plane elasticity modulus measured using Langmuir trough technology (compare Figure 4 from Reference Nencheva et al.^55^ and Figure 11E from Borchman^20^ with Figures 11A–11D in Borchman^20^). As a consequence of less HCunsat, meibum lipids become more ordered or stiff, which could inhibit the flow of meibum from the meibomian glands. It is reasonable to speculate that these more ordered lipids contribute to the formation of a discontinuous, patchy (more heterogeneous) TFLL. This alteration in the structure of the TFLL is associated with deteriorated spreading and surface elasticity of the tear film. It is an attractive hypothesis that, as HCunsat, lipid order, and elasticity increase with age in individuals without DED, a threshold may be reached above which dry eye occurs.

No differences in the HCunsat were measured in the current study between males and females, suggesting that HCunsat age-related differences related to sex are not involved with sex-related susceptibility to DED.^56^^,^^57^ It is notable that Hispanics had a lower HCunsat compared with Caucasians; however, this result should be viewed with caution owing to the limited number of samples.

HCunsat was measured in the current study for a limited number of participants with SS, PD, and AHSCT. As observed in the current study, people with SS and PD, who are susceptible to DED, have a lower level of HCunsat, suggesting that HCunsat may contribute to lower tear breakup times and higher blink rates.^20^ Lipid order and phase transition temperature were slightly higher for meibum from C_PD_ compared with Cn.^37^ Lipid order and phase transition temperatures were especially higher for meibum from C_PD_ donors with DED compared with Cn and C_PD_ without DED, in accordance with a higher level of HCunsat.^37^ The lower level of HCunsat measured in the current study for participants with SS did not manifest in a higher level of lipid order.^36^ HCunsat was 32% lower for C_AHSCT_ compared with Cn, likely causing the increase in lipid order observed for this cohort compare with Cn.^20^^,^^39^^,^^40^ C_AHSCT_ did have 49% less CE compared with Cn,^20^^,^^39^^,^^40^ which could have influenced the measurement of HCunsat, measured in the current study, because the HCunsat was measured relative to the levels of WE and CE. Numerous resonances, especially at 2 ppm, were observed in the ^1^H-NMR spectra from C_AHSCT_ most likely involving breakdown products from radiation treatment.

The variability in HCunsat collected from a single donor and measured once, then five times 1 week apart after 15 years, was only 3% to 4%, a unique finding! Thus, the variability observed in HCunsat with age from multiple individuals in the current study is most likely due to individual variability and not due to experimental deviation.

### Advantages of the Spectroscopic Measurement of HCunsat

The advantages and power of spectrometric lipidomic techniques include their ability to identify and quantify specific tear and meibum lipid moieties, a major advantage over the spectroscopic techniques used in the current study. However, complete quantification using spectrometric techniques is complicated by the great number (hundreds) of species present in human meibum.^58^ Lack of specificity of spectroscopic techniques can be an advantage. For instance, all double bonds provide a ^1^H-NMR resonance at 2 ppm, regardless of whether they come from WE, CE, or numerous other moieties in meibum. To measure the HCunsat using ^1^H-NMR spectroscopy, one only needs to measure the intensity of four, well-resolved resonances (Equation 1). HCunsat can be calculated without the need to know the molecular weights of the moieties by accounting for the stoichiometry of the hydrogen resonances, 2 mol from the WE at 4.0 ppm and 6 mol from the CE at 1.0 and 0.63 ppm and 4 mol from the double bond resonance at 2 ppm. To measure the HCunsat by spectrometric measurements, one must identify and quantify all the hundreds of species that are associated with double bonds, know their molecular weights, and sum the number of species with double bonds to be compared with the sum of all the CE and WE esters. Also, because each lipid class has a different ionization efficiency or response factor, standards are needed for the spectrometric quantification of each of the hundreds of moieties in meibum. As stated, standards are not needed for quantification using ^1^H-NMR spectroscopy. Furthermore, the sample is not destroyed using spectroscopic measurements, so the same sample could be used for spectrometric measurements, other spectroscopic techniques, or rheological studies.

A major contribution of the current study is that meibum unsaturation was measured in 239 samples, the largest number of samples studied related to unsaturation. Patient age was approximately 50 years.^22^ The current study analyzed 139 samples, 118% more than the previous study.^22^ The largest number of samples studied related to unsaturation and dry eye was 41.^27^ The current study analyzed for the first time, 68 samples with MGD, 66% more than the previous study,^27^ and 32 additional samples with maladies associated with dry eye. An advantage of the current study is that the 2 ppm resonance as well as the 5.33 resonance were used to measure HCunsat in contrast with just using the 5.33 ppm resonance.^22^ The 2 ppm resonance is well-resolved in the ^1^H-NMR spectra of human meibum, unlike the resonance at 5.35 ppm that overlaps with resonances from CE previously used to measure HCunsat associated with age^22^ and four times more intense (less noise) than the 5.35 ppm resonance used previously. The level of unsaturation measured using the 2 ppm resonance in the current study was measured relative to the total amount of esters or total amount of methylene moieties, an advantage over the previous study.^22^ Another major advantage of the current study, over our previous study,^22^ is that, in the current study, resonances at 1.0 and 0.63 ppm were used to measure the amount of CE. These resonances were six times more intense and contained less noise than the CE resonance at 4.6 ppm used in the previous study.^22^

An advantage of the current study over our previous study^27^ is that all of the meibomian glands were expressed in the current study, compared with only five glands expressed in the previous study. The amount of meibum collected in the current study was more than eight times greater than that of the previous study,^27^ providing a better quality ^1^H-NMR spectra.

Studies exploiting the advantages of the spectroscopic techniques used in the current study are being prepared highlighting differences in the levels of HCunsat, CE, WE, and (O-acyl)-ω-hydroxy fatty acids in cohorts with different types of dry eye, C_SS_, C_PD_, and C_AHSCT_. A study is also underway to measure the influence of compositional changes using molecular dynamics simulations.

## Conclusions

Meibum HCunsat decreases with age, as well as in DED, SS, and PD cohorts, compared with the Cn. This decrease in HCunsat is expected to aggregate lipids, decrease lipid spreading, and lower lipid outflow from the meibomian glands, all of which would be expected to contribute to lower breakup times and higher blink rates. The ^1^H-NMR spectroscopic technique provides a powerful, nondestructive way to quantify these changes.
